# Mitochondrial mutations and metabolic adaptation in pancreatic cancer

**DOI:** 10.1186/s40170-017-0164-1

**Published:** 2017-01-30

**Authors:** Rae-Anne Hardie, Ellen van Dam, Mark Cowley, Ting-Li Han, Seher Balaban, Marina Pajic, Mark Pinese, Mary Iconomou, Robert F. Shearer, Jessie McKenna, David Miller, Nicola Waddell, John V. Pearson, Sean M. Grimmond, Australian Pancreatic Cancer Genome Initiative, Leonid Sazanov, Andrew V. Biankin, Silas Villas-Boas, Andrew J. Hoy, Nigel Turner, Darren N. Saunders

**Affiliations:** 1 The Kinghorn Cancer Centre, Garvan Institute of Medical Research, Darlinghurst, NSW 2010 Australia; 2 St Vincent’s Clinical School, University of New South Wales, Sydney, NSW Australia; 3 School of Biological Sciences, University of Auckland, Auckland, 1142 New Zealand; 4 Discipline of Physiology, School of Medical Sciences and Bosch Institute, University of Sydney, Sydney, NSW 2006 Australia; 5 Centre for Medical Genomics, Institute for Molecular Bioscience, University of Queensland, St. Lucia, QLD 4072 Australia; 6 Mitochondrial Biology Unit, Wellcome Trust, Cambridge, CB2 0XY UK; 7 Wolfson Wohl Cancer Research Centre, Institute of Cancer Sciences, University of Glasgow, Glasgow, UK; 8 Boden Institute of Obesity, Nutrition, Exercise and Eating Disorders, University of Sydney, Sydney, NSW 2006 Australia; 9 School of Medical Sciences, University of New South Wales, Sydney, NSW 2052 Australia

**Keywords:** Pancreas, Mitochondria, Metabolomics, Glutamine, Lipid, Genome

## Abstract

**Background:**

Pancreatic cancer has a five-year survival rate of ~8%, with characteristic molecular heterogeneity and restricted treatment options. Targeting metabolism has emerged as a potentially effective therapeutic strategy for cancers such as pancreatic cancer, which are driven by genetic alterations that are not tractable drug targets. Although somatic mitochondrial genome (mtDNA) mutations have been observed in various tumors types, understanding of metabolic genotype-phenotype relationships is limited.

**Methods:**

We deployed an integrated approach combining genomics, metabolomics, and phenotypic analysis on a unique cohort of patient-derived pancreatic cancer cell lines (PDCLs). Genome analysis was performed via targeted sequencing of the mitochondrial genome (mtDNA) and nuclear genes encoding mitochondrial components and metabolic genes. Phenotypic characterization of PDCLs included measurement of cellular oxygen consumption rate (OCR) and extracellular acidification rate (ECAR) using a Seahorse XF extracellular flux analyser, targeted metabolomics and pathway profiling, and radiolabelled glutamine tracing.

**Results:**

We identified 24 somatic mutations in the mtDNA of 12 patient-derived pancreatic cancer cell lines (PDCLs). A further 18 mutations were identified in a targeted study of ~1000 nuclear genes important for mitochondrial function and metabolism. Comparison with reference datasets indicated a strong selection bias for non-synonymous mutants with predicted functional effects. Phenotypic analysis showed metabolic changes consistent with mitochondrial dysfunction, including reduced oxygen consumption and increased glycolysis. Metabolomics and radiolabeled substrate tracing indicated the initiation of reductive glutamine metabolism and lipid synthesis in tumours.

**Conclusions:**

The heterogeneous genomic landscape of pancreatic tumours may converge on a common metabolic phenotype, with individual tumours adapting to increased anabolic demands via different genetic mechanisms. Targeting resulting metabolic phenotypes may be a productive therapeutic strategy.

**Electronic supplementary material:**

The online version of this article (doi:10.1186/s40170-017-0164-1) contains supplementary material, which is available to authorized users.

## Background

Pancreatic cancer has one of the worst survival rates, with fewer than 8% of patients surviving 5 years post-diagnosis [1]. A near universal feature of pancreatic cancer, and one of its earliest molecular changes, is a constitutively activating oncogenic KRAS mutation [2–5]. Along with established roles in driving cell proliferation and survival, KRAS and several other oncogenes (e.g., AKT) and tumour suppressors (e.g., TP53), have recently been shown to regulate metabolic pathways in pancreatic and other cancer cells [6–8]. These metabolic changes include increased use of glutamine to support cell growth and proliferation, increased NADPH/NADP^+^ ratio to maintain cellular redox state [9] and rewiring anabolic glucose metabolism by inducing glucose uptake and enhancing glycolysis [10].

The role of altered nutrient metabolism in cancer cells has attracted significant renewed interest, both in understanding tumourigenesis and as a potential therapeutic target [6, 11–13]. Tumour cells upregulate the production of biosynthetic intermediates for the building of new cells, while maintaining or even slightly increasing ATP levels and energy production. Some of the emerging hallmarks of cancer metabolism include, but are not limited to use of opportunistic modes of nutrient acquisition and use of glycolysis/TCA cycle intermediates for biosynthesis and NADPH production [14]. Glycolytic intermediates are shunted into auxiliary pathways, driving generation of nucleotides, lipids, amino acids, and complex sugars [15–17]. Cancer cells also use complementary fuel sources (e.g., amino acids and fatty acids), and proliferating cells have increased uptake of these metabolic substrates to meet increased demands for biosynthesis and energy production. For example, glutamine can be used in place of glucose to fuel the TCA cycle, sparing glucose for glycolytic biosynthesis of cellular building blocks [18]. The mechanisms underlying these metabolic shifts in different cancer types are not fully established, and are likely complex given the highly integrated nature of genes and signalling pathways regulating metabolism [17]. Targeting metabolism may be an effective therapeutic strategy for cancers that are driven by genetic alterations that are not tractable as direct drug targets [11, 19]. In the context of the very high penetrance of KRAS mutations in pancreatic cancers, targeting metabolic enzymes was effective in treating KRAS-mutant tumours in pre-clinical lung cancer models [12].

Mitochondrial dysfunction and mutations in mitochondrial genes have been implicated in shifting cellular metabolism to a state favouring tumour proliferation [20–22]. While cells completely devoid of mtDNA (rho-0) have reduced tumorigenic and metastatic capacity in mice [23, 24] and need to acquire mtDNA from host cells to restore function and growth [25], partial mitochondrial dysfunction is known to induces migration, invasion, and metastasis [24].

**Fig. 1:**
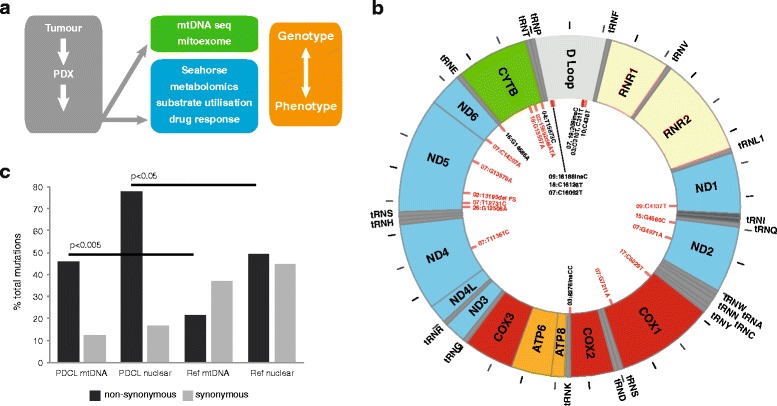
Mitochondrial genome (mtDNA) sequence analysis in pancreatic cancer PDCLs. **a** Schematic showing the approach used to map genotype:phenotype relationships in pancreatic cancer. **b** Distribution of somatic mtDNA mutations (*red lines*, *n* = 24) in 12 pancreatic PDCLs, showing strong bias towards variants in ETC complex I coding and control regions. ETC subunit coding regions are denoted by subunit (*colour coded* by ETC complex). Position of tRNAs are noted, with ticks marking 500 bp intervals. **c** Strong selection for non-synonymous mutants in mtDNA and 1056 nuclear genes important for mitochondrial function and metabolism in pancreatic PDCLs compared with a reference survey of mitochondrial variants in infantile mitochondrial disease (Calvo et al. [39]). Statistical comparisons performed using Chi-squared analysis

Accumulation of somatic mutations in the mitochondrial genome (mtDNA) have been observed in various tumour types [26, 27], but common “driver” mutations have not been clearly identified and a limited number of studies have shown a direct role for specific mtDNA mutations in tumourigenesis using mitochondrial cybrid models [28, 29]. In short, there is limited understanding of genotype-phenotype relationships, or how these mutations precisely impact on mitochondrial function and cellular metabolism. The availability of a cohort of patient-derived cell lines (PDCLs) through the Australian Pancreatic Cancer Genome Initiative (APGI) [30, 31] presents a unique opportunity to directly study links between genotype and phenotype in pancreatic cancer (Fig. 1a). This study is the first to use patient derived cell lines to connect genotype to phenotype in pancreatic cancer by measuring metabolic function in the context of somatic mutations.

## Methods

### Patient-derived xenograft (PDX) generation

Six-to-eight-week-old female NOD/SCID/IL2Rgamma^-/-^ (NSG) mice and athymic Balb-c-nude mice were used for the establishment of the patient derived xenograft (PDX) model. All mice were bred at the Australian Bioresources (ABR). PDX were generated using a modified version of the methodology published [32–34]. Briefly, surgical non-diagnostic specimens of patients operated at the various APGI clinical sites were implanted subcutaneously into three NSG and three Balb-c-nude mice for each patient, with two small pieces per mouse (left and right flank; engraftment stage). Once established, tumours were grown to a size of 1500 mm^3^, at which point they were harvested, divided, and re-transplanted into further mice to bank sufficient tissues for experimentation (first passage and second passage). After expansion, passaged tumours were excised and propagated to cohorts of 40 mice or greater, which constituted the treatment cohort (third passage). Utilisation of the NOG mouse model, which is characterized by high immune deficiency in this study has enabled establishment of a significant cohort of PDXs (70) xenografts, with a high rate of successful engraftment and propagation (76%, data not shown).

### Patient-derived cell line (PDCL) generation

The selected patient-derived cell lines (PDCL) used in this study, named The Kinghorn Cancer Centre (TKCC) lines, were established by plating and growing cells from an enzymatically digested xenograft on a collagen matrix for approximately 1 week prior to removal of fibroblasts from the mixture using flow cytometry with anti-mouse CD140a-PE (BD Biosciences, USA) and anti-mouse MHCI-H_2_K_D_ antibody, eBiosciences, USA) (Pajic et al., *manuscript in preparation*). All cell lines were profiled by short tandem repeat (STR) DNA profiling as unique (CellBankaustralia.com).

### Cell culture procedures

Human pancreatic ductal endothelial (HPDE) cells were used as a normal pancreatic cell control [35]. These cells were routinely cultured in keratinocyte serum-free (KSF) medium supplemented by epidermal growth factor and bovine pituitary extract (Gibco Life Technologies USA). After patient-derived xenografts (PDX) were completed, PDX-derived PDCLs were routinely cultured in conditions specifically formulated for each individual cell line (refer Additional file 1: Supplementary Methods). In the generation of each cell line (as described in [4, 34], different media, culture conditions, and levels of oxygen (both normal culture conditions and low, 2% which is physiologically close to the oxygen levels found in the hypoxic centre of a tumour) were tested. In vitro growth conditions were selected based on conditions resulting in best cell growth through multiple passages and those resulting in cell lines most closely resembling physiological cells from the initial tumour.

### Mitochondrial genome sequencing

Mitochondrial genomes of each cell line (PDCLs, HPDE and HPDE-KRAS^G12V^), along with matched normal DNA from each patient, and tumour DNA where available, were sequenced a modified version of the protocol described by [36]. Briefly, two overlapping long-range PCR amplifications were performed, generating 7.2 Kb Amplicon 1 (spanning bp 12,256-3,005) using primers Amp1-F: 5′-GGCTTTCTCAACTTTTAAAGGATA-3′ and Amp1-R: 5′-TGTCCTGATCCAACATCGAG-3′; and 9.7 Kb Amplicon 2 (spanning bp 2,583-12,337) using primers Amp2-F: 5′-CCGTGCAAAGGTAGCATAATC-3′ and Amp2-R: 5′-TTACTTTTATTTGGAGTTGCACCA-3′. Platinum Taq polymerase kit (Invitrogen) was used and touchdown cycling was performed as follows: (1) 94 °C for 3 min, then (2) 10 cycles of 94 °C for 30 s, 71 °C for 45 s (decrease by 1 °C per cycle), 68 °C for 8 min, then (3) 25 cycles of 94 °C for 30 s, 61 °C for 45 s, and 68 °C for 8 min, and finally (4) 68 °C for 5 min, 4 °C for 15 min, and 10 °C hold. PCR cleanup was performed using 0.6 μl Exonuclease I, 3 μl SAP, 11.4 μl nuclease free water, and 30 μl PCR product, incubated at 37 °C for 75 min, 80 °C for 20 min, then cooled to 4 °C. Sanger sequencing was performed using 43 internal sequencing primers (refer Additional file 1: Supplementary methods) using the two large amplicons as template.

mtDNA sequence chromatogram base calls, alignments and variant calls were performed using Sequencher software, with manual verification. Overlapping sequences were aligned against the linearised revised Cambridge reference sequence (rCRS) starting at position 1. When overlapping sequences crossed the D-loop and position 1, we ensured this sequence was represented at both ends of the linearised sequence to cover all bases. mtDNA sequences have been deposited in the European Nucleotide Archive (ENA) with study accession number: PRJEB18798.

### Nuclear genome sequencing

The Australian Pancreatic Cancer Genome Initiative (APGI) undertook the prospective recruitment of a cohort of early stage (non-metastatic), non pre-treated pancreatic ductal adenocarcinomas. At the time of surgery, primary tumour specimens are obtained, in addition to matched normal tissue, from the duodenum. High confidence somatic single nucleotide variants and small insertions and deletions were identified from exome-capture sequencing using SOLiD v4 from primary tumours as previously reported [31], or from patient derived xenografts and cell lines, sequenced using Illumina HiSeq 2000, using qSNP [37] and Pindel [38], respectively, as described in [31].

For the identification of nuclear encoded mitochondrial genes that are mutated in pancreatic cancer, the published list of 1034 genes [39], was supplemented with 22 additional genes that have been implicated in mitochondrial function (Additional file 1: Table S1). These mixed gene symbols and aliases were updated to official gene symbols using custom scripts leveraging the org.Hs.eg.db and AnnotationDbi Bioconductor packages, and NCBI mapping data obtained 4/9/2012. Somatic mutation data from primary tumours were used for 5/12 of the cell lines studied here [31], while for the remaining 7/12 cell lines mutations were identified from HiSeq 2500 data comparing xenograft versus normal (*unpublished*). Mutations are reported for nuclear encoded mitochondrial genes in mutation annotation format (MAF) file.

### Cellular bioenergetics measurements

Cellular oxygen consumption rate (OCR) and extracellular acidification rate (ECAR) were measured with a Seahorse XF extracellular flux analyser (Seahorse Bioscience Inc) according to the manufacturer’s instructions. Briefly, cells were seeded in a Seahorse XF 24-well assay plate at a cell density between 15,000 and 30,000 cells per well in full growth medium. After overnight attachment, the medium was washed and replaced with prewarmed running medium (non-buffered DMEM supplemented with 1 mM sodium pyruvate and 10 mM glucose, pH 7.4) and incubated in a non-CO2 incubator at 37 °C for 60 min. Basal levels of OCR and ECAR were recorded for 24 min, followed by a mitochondrial stress test (1 μg/ml oligomycin, 0.3 μM FCCP, 1 μM rotenone/1 μM antimycin A) [40]. Cells were lysed post-measurement and protein content estimated using BCA Assay (Pierce).

### Metabolomics

Preparation of samples followed a modified version of protocol described by [41], optimised for human cells. Cells were quenched and washed with 0.9%w/v NaCl at 4 °C and metabolites were extracted by the addition of cold 50% methanol. 20 μl of d4-alanine (Sigma-Aldrich) was added to each sample (10 mM final) as an internal control. Samples were subjected to four rounds of vortexing and freeze-thawing (−80 °C for ~30 min) before centrifugation at 15,000 rpm for 15 min at -20 °C. Supernatant was decanted and stored at -80 °C. Remaining cell pellet was subjected to sequential methanol extraction using 80 and 100% methanol, vortexing and centrifugation as above. Extractions were pooled and deionised water added to bring methanol concentration below 20% before being frozen at −80 °C and lyophilised. Analysis was focused on amino acids, organic acids and fatty acids (Additional file 1: Table S3) using 20–25 mg of cell dry-weight per sample. Samples were derivatised using methyl chloroformate and analysed by gas chromatography–mass spectrometry (GC–MS) [41]. Pathway activity profiling (PAPi) was used to compare metabolic pathway activities from metabolic profiles [41, 42]. Samples were normalised by the abundance of internal standard (2,3,3,-d_4_-alanine) and biomass content (Additional file 2: Table S4). Activity scores were assigned to each pathway, based on the number and abundance of the relevant metabolites identified within the samples of this study. Partial least squares discrimination analysis (PLS-DA) of all pancreatic cell lines was performed using Multibase (http://www.numericaldynamics.com). ANOVA, Tukey’s HSD and hierarchical clustering were performed to determine which pathways were activated or attenuated in PDCLs compared with HPDE. Heat map representations of the results were produced by ggplot2 R packages [43].

### Radiolabelled glutamine tracing

The rate of conversion of glutamine to lipid was measured by incubating PDCLs in DMEM containing 2% BSA, 5 mM glucose, 0.5 mM oleate, 0.5 μCi/ml ^14^C L-glutamine, 2 mM L-glutamine and 1 mM carnitine for 4 h. Cells were rinsed in PBS and lipids extracted in chloroform:methanol (2:1 v:v). Following centrifugation (1000 g for 10 min) the organic phase was evaporated to dryness under nitrogen gas at 40 °C and radiation measured by scintillation counter (1900CA Tri-Carb Liquid Scintillation Analyzer, Packard).

## Results

### Novel somatic mitochondrial DNA mutations in pancreatic PDCLs

While extensive genomic profiling of pancreatic cancer has been performed by us and others [4, 5, 31, 44–46], these analyses did not report mtDNA sequences. Hence, mtDNA sequencing in twelve PDCLs and matched normal DNA from each patient in the APGI cohort [31] identified 24 somatic mutations (Fig. 1b and Table 1). Significantly, these mutations occurred mainly in coding regions of ETC Complex I and control regions, and the majority of coding region variants observed were non-synonymous. Comparison with MitoMap [47] indicates that most of the mutations identified have not been previously described, particularly in a cancer context.

**Table 1:** Somatic mtDNA mutations identified in 12 PDCLs

Cell line	ChrM position	Normal	Tumour	Cell line	AA change	Region	ETC complex	Literature
TKCC-02	13,195–14,000	cons	2 bp del	2 bp del	Frameshift	ND5	I	Similar region- colorectal cancer: Polyak 1998
TKCC-03	310	C/T	T	T	-	HVSII	-	
	311	C/T	C	C	-	HVSII	-	
	8276.3–8276.4	CC ins	::	::	-	NC7	-	
	15,692–15,694	ATA	::: del	::: del	M316Δ	CytB	III	T15693C LVNC cardiomyopathy: Tang 2010
TKCC-04	15,873	T	-	C/T	M376T	CytB	III	NOVEL
TKCC-07	309.3	:	-	C ins	-	HVSII	-	Alzheimer’s: Tanaka 2010
	4971	G	-	A	G168S	ND2	I	NOVEL
	7211	G	-	A	M436M	COI	IV	
	11,361	T	-	C	M201T	ND4	I	
	12,731	T	-	C	V132A	ND5	I	NOVEL
	13,579	G	-	A	A415T	ND5	I	NOVEL
	14,207	G (C reverse)	-	A (T reverse)	T156I	ND6	I	
	16,092	C	-	T	-	HVSI	-	
TKCC-09	4137	C	T	T	Y277Y	NDI	I	
	16,188.1	:	:	C ins	-	HVSI	-	
TKCC-10	438	C	C/T	T	-	L strand promoter	-	
TKCC-15-LO	4560	G	-	C/g	W30S	ND2	I	NOVEL
TKCC-16-LO	14,686	G	-	G/a	-	TE (tRNA)	-	NOVEL
TKCC-17-LO	6029	C	-	T/c	G42G	COI	IV	
TKCC-18-LO	15,557	G	-	A	Q271K	CytB	III	NOVEL
	16,126	C	-	T (wt ref. seq)	-	HVSI	-	Glioblastoma T > C Kirches 2001, Brandon 2006
TKCC-19-LO	309.1–309.2	C	CC	CC	-	HVSII	-	
TKCC-26-LO	12,508	G	-	A	P58N	ND5	I	NOVEL

All PDCLs sequenced (based on availability of matched normal DNA for comparison) had at least one mutation present, with eight mutations in TKCC-07. While the same base pair mutation was not observed repeatedly, multiple mutations were found in common ETC subunits across different cell lines. Most mutations identified were either non-synonymous, and predicted to result in amino acid changes (11/24, 46%) or located in control regions (10/24, 42%). Only 12.5% (3/24) of mtDNA mutations were synonymous or silent mutations. Comparison with a reference survey of mitochondrial variants in infantile mitochondrial disease [39] indicates that there is a very strong positive selection bias for non-synonymous variants in coding regions of the mtDNA in pancreatic tumours (Fig. 1c).These data are strongly suggestive of positive selection bias for mutations causing functional effects via amino acid changes or transcriptional control)

The majority of somatic mtDNA mutations in PDCLs (summarised in Fig. 1b) were observed in ETC complex I subunits (*n* = 9), and noncoding control regions (*n* = 10) such as the hypervariable segments located in the D-loop, NC7, L promoter, and tRNA glutamic acid. Somatic mutations were distributed across five of the seven complex I subunits that are encoded by the mtDNA. Somatic mutations were also seen in cytochrome C oxidase subunit I (COI of complex IV (*n* = 2)) and cytochrome B (CyB) of complex III (*n* = 3).

In a few rare cases, DNA samples from the original tumour were available, and corresponded with the sequence of the tumour cell lines. For example, TKCC-09 and TKCC-19-LO had mutations in both the tumour and tumour cell line. Of the 24 cell line mutations, 4 were heteroplasmic and 20 were homoplasmic. One interesting example is TKCC-03, where both tumour and cell line were homoplasmic for T and C at bp 310 and 311 respectively, but matched normal tissue was heteroplasmic at these sites (i.e., both C and T were present at each site). In TKCC-10, heteroplasmy was observed in the tumour at base pair 438 (C/T) while the cell line was homoplasmic for this mutation (C > T) (Additional file 1: Figure S1). This could indicate either improved tumour cell purity in the genetic sample from the PDCL, the presence of contamination of normal tissue in the surgical tumour sample, or positive selection for the mutation under culture conditions. Heteroplasmy was also observed in TKCC-15-LO, -16-LO, and -17-LO, with levels of the mutant higher than the wild type sequence in all cases. TKCC-02 harbours a deletion resulting in a frameshift mutation in a region of ND5 (Complex I), which has been implicated in colorectal cancer [48]. Interestingly, mtDNA sequence of a liver metastasis from the patient corresponding to TKCC-19-LO showed that the metastasis had the identical sequence as the original tumour, with the same somatic mutation (chrM:g.3091-3092insC). Of note, no mutations were detected in the mtDNA of HPDE after extended culture following transfection with mutant KRAS (G12D).

### Functional effects of somatic mtDNA mutations predicted by molecular modelling

To predict functional effects of non-synonymous mutations, molecular modelling was performed using a human homology model of the structure of ETC complex I (based on PDB entry 4HEA) [49] (Table 2). All mutations were located in the membrane domain of the complex, and were predicted to affect complex assembly (due to stability and protein folding issues), enzyme activity, and/or proton leakage. Modelling was also performed on ETC complex III mutations (bovine model, PDB entry 2FYU) [50], with predicted effects on its cytochrome B subunit including possible changes in redox capacity (Table 2).

**Table 2:** Predicted effects of mutations in ETC subunits

ETC subunit	PDCL	Mutation	Predicted effect
Complex I human residues (corresponding *Thermus thermophilus* residues)			
ND5	TKCC-02	Frameshift	Part of ND5 loss, therefore no active complex I
	TKCC-07	V132A (V119)	V sits at the end of TM4 and faces TM3, may slightly destabilise fold in this area.
	TKCC-07	A415T (V409)	A sits on TM13, facing TMs 9 and 10, may de-stabilise fold in this area and/or make channel cavity leaky in this area.
	TKCC-26-LO	D58N (F55)	D sits on the beta-sheet from the beta-H motif, may interfere with conformational coupling.
ND4	TKCC-07	M201T (L199)	M sits on key flexible helix 7 and faces the lipid or supernumerary subunit, may interfere with mechanics of TM7 and decrease NADH:Q oxidoreductase activity and/or proton pumping.
ND2	TKCC-07	G168S (A247)	Directly facing traverse helix HL from ND5, might decrease NADH:Q oxidoreductase activity (or proton pumping only) preventing full movement of HL.
	TKCC-15-LO	W30S (L108)	W provides part of a seal between ND2 and ND4L, therefore mutation might make it somewhat leaky to protons.
ND6	TKCC-07	T156I (V142)	T is near key ND2 Glu34 (GluTM5), so the mutation may interfere with its pKa and so with proton pumping through ND2. Fold in this area will also be disturbed.
Complex III (bovine model 2FYU)			
CytB subunit	TKCC-03	M316Δ	May interfere with fold at the junction with subunit 6.
	TKCC-04	M376T	Leu in bovine- peripheral, but may interact with N-term helix from subunit 7.
	TKCC-18-LO	E271K	E in bovine- close to heme (6 A, directly facing the edge), so may well interfere with its redox properties.

*TM* transmembrane, *ND* NADH dehydrogenase subunit, *CytB* cytochrome B

### Somatic nuclear mutations implicated in mitochondria and metabolism

As part of the APGI project, nuclear genome sequence data for the PDCL cohort was also available [4, 5, 31]. Calvo et al. previously created a comprehensive list of 1034 nuclear genes encoding mitochondrial proteins, as part of the “Mitoexome” in their study of infantile mitochondrial disease [39]. These genes, along with 22 additional genes that have been implicated in metabolic function, were analysed for somatic mutations in primary tumours corresponding to PDCLs (complete list of genes analysed is provided in Additional file 1: Table S1). In addition to mtDNA mutations, a total of 18 somatic nDNA mutations were discovered in this targeted mitochondrial/metabolic gene set (in 16 genes, 2 were in TP53 and 2 in ADCK4) (Table 3). Notably, mutations were not observed in nuclear-encoded ETC subunits. Consistent with the genetic pathology of pancreatic cancer, all patient samples also had somatic KRAS mutations [3]. As observed for mtDNA mutations, comparison with mitochondrial variants in infantile mitochondrial disease [39] indicated strong positive selection bias for non-synonomous variants (Fig. 1b).

**Table 3:** Somatic nuclear DNA mutations in nuclear encoded mitochondrial and metabolic genes in TKCC pancreatic tumours (*n* = 12)

Cell line	Gene	Chrom	Position	Variant Class	Ref. Allele	Tumour Allele 1	Tumour Allele 2	dbSNP RS	Normal Allele 1	Normal Allele 2	Bp change	AA change
TKCC-02	TRIT1	1	40319722	Nonsense	G	A	G	novel	G	G	G > A	R112*
TKCC-03	None detected											
TKCC-04	MRPS21	1	150266837	Silent	G	C	G	novel	G	G	113G > C	G17G
	TP53	17	7578406	Missense	C	T	T	rs28934578	C	C	524C > T	R175H
	ACACA	17	35615179	Missense	C	A	C	novel	C	C	2099C > A	R539S
	CPT1C	19	50203981	Missense	G	A	G	novel	G	G	694G > A	A108T
	PIK3CA	3	178952085	Missense	A	A	T	rs121913279	A	A	3297A > T	H1047L
	PDHA2	4	96761551	Missense	T	C	T	novel	T	T	313 T > C	F84L
TKCC-07	None detected											
TKCC-09	AKT3	1	243736250	Missense	T	C	T	novel	T	T	863 T > C	K266R
	YME1L1	10	27437884	Missense	T	G	T	novel	T	T	301 T > G	Q40P
	ADCK4	19	41208536	Missense	G	A	A	novel	G	G	1164G > A	R288C
TKCC-10	None detected											
TKCC-15-LO	MTCP1	X	154293944	Missense	G	A	G	novel	G	G	G > A	R76C
TKCC-16-LO	HMGCL	1	24143254	Missense	C	A	C	novel	C	C	303C > A	D87Y
	ADCK4	19	41198257	Missense	C	C	G	novel	C	C	1620C > G	E440Q
	AGXT2	5	35013131	Silent	C	C	T	novel	C	C	1317C > T	A327A
TKCC-17-LO	TP53	17	7578553	Splice Site	T	C	C	novel	T	T	377 T > C	Y126C
TKCC-18-LO	TARS2	1	150477113	Missense	C	C	T	novel	C	C	C > T	A575V
TKCC-19-LO	None detected											
TKCC-26-LO	LDHB	12	21788484	Missense	C	A	C	novel	C	C	C > A	D333Y
	ADSL	22	40676075	Silent	G	A	G	novel	G	G	G > A	

Somatic mutations were identified in several key genes involved in metabolism (Table 3 and Additional file 1: Table S2). These include two enzymes in fatty acid metabolism: Acetyl Co-A carboxylase (ACACA), involved in the rate limiting step of fatty acid synthesis and carnitine palmitoyltransferase I (CPT1C), an acetyltransferase which transports long chain fatty acids across the outer mitochondrial membrane. Mutations were also found in pyruvate dehydrogenase alpha 2 (PDHA2) which catalyses oxidative decarboxylation of pyruvate as well as lactate dehydrogenase B, catalysing reversible conversion of lactate to pyruvate. While some PDCLs contain few somatic mutations in mtDNA and several in nuclear DNA (e.g., TKCC-04 and TKCC-09), TKCC-07 had no nDNA mutations but many in the mtDNA. In summary, there is a possibility for mixed effects of mtDNA and nuclear mutations.

### Metabolic phenotype of pancreatic tumour cell lines

**Fig. 2:**
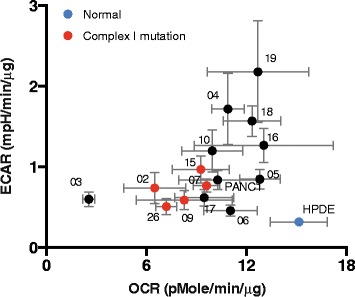
Metabolic profile of TKCC and normal HPDE pancreatic cell lines. Basal oxygen consumption rate (OCR) and extracellular acidification rate (ECAR) during mitochondrial stress test using Seahorse analyser (data presented as mean +/− s.d., *n* = 5)

Mitochondrial and glycolytic stress tests were performed to measure oxygen consumption rate (OCR) and extracellular acidification rate (ECAR) of PDCLs, as approximates of mitochondrial respiration and glycolysis, respectively. Human pancreatic ductal epithelial (HPDE) cells were used as a control as they are the closest available to the normal cells from which pancreatic tumours in this study were derived [35, 51–53]. Comparing overall metabolic profiles (OCR vs ECAR) for 16 tumour cell lines and HPDE control cells (Fig. 2) showed that PDCLs have relatively lower OCR and higher ECAR. This is consistent with the concept of aerobic glycolysis (Warburg effect) in tumour cells, with increased ECAR indicating higher glycolytic flux. Lower OCR in PDCLs indicates attenuated oxidative phosphorylation in these cells. In the context of somatic mtDNA mutations in these cells, this effect most likely reflects defects in mitochondrial respiration. Notably, those PDCLs harbouring mutations in ETC complex I (TKCC-02, TKCC-07, TKCC-09, TKCC-15-LO, TKCC-26-LO), complex III (TKCC-03), or complex IV (TKCC-07, TKCC-17-LO) showed the largest attenuation of oxygen consumption relative to HPDE, supporting the predicted functional effects of somatic mtDNA mutations on ETC function (Table 2).

### Metabolomic profiling of pancreatic cancer PDCLs

**Fig. 3:**
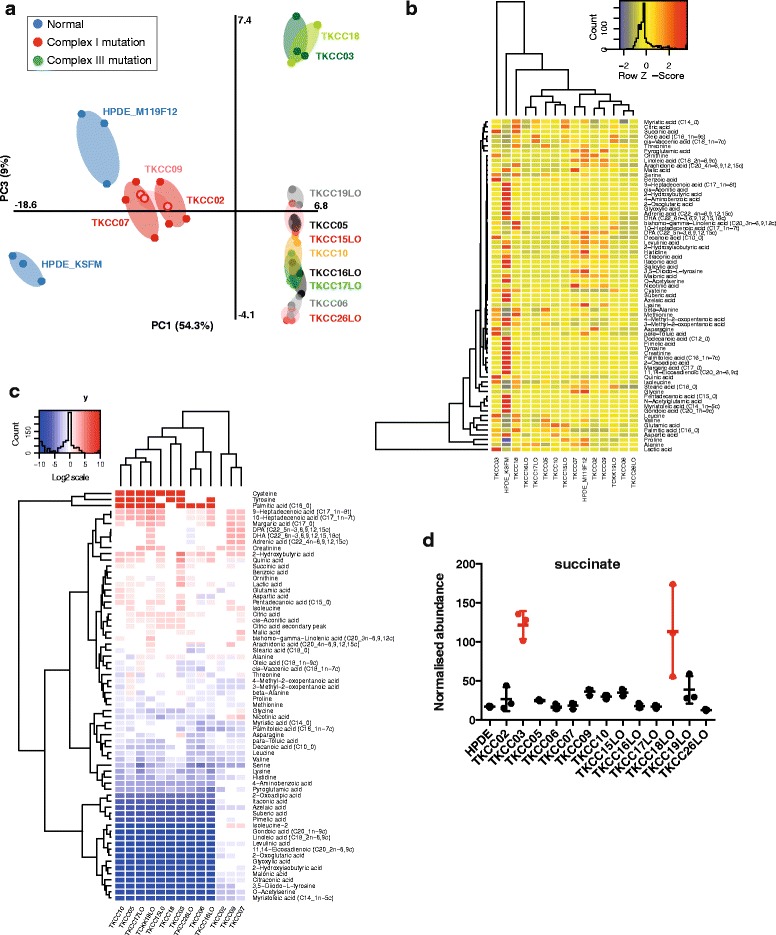
Metabolomics analysis of pancreatic cancer PDCLs. **a** Partial least squares discrimination analysis (PLS-DA) of metabolomics profiles from all pancreatic cell lines (normal and PDCL) used in this study. **b** Relative intracellular metabolite levels for 13 pancreatic PDCLs and normal HPDE pancreatic cell line grown in two different media (K-SFM and M199/F12). Colour key (*top right*) is superimposed by a histogram showing counts of all identified metabolites (Z-score). *Rows* list the identified metabolite names, and columns list the pancreatic cell lines arranged according to hierarchical clustering analysis. **c** Heirarchical clustering analysis of changes in intracellular metabolite levels for 13 pancreatic PDCLs relative to HPDE normal cells (in K-SFM media). Fold change of metabolites with significantly different levels by ANOVA (*p* < 0.05) are shown. **d** Succinate abundance in PDCLs and HPDE (normalised to protein content)

We deployed a targeted metabolomics approach to determine changes in metabolic flux of pancreatic cancer PDCLs using a list of 72 metabolites representing major metabolic pathways (Additional file 1: Table S3). Relative abundance of each metabolite was determined following normalisation to protein content. Partial Least Squares Discrimination Analysis (PLS-DA) of metabolite profiles of each of the 13 PDCLs and normal HPDE cells (Fig. 3a) showed that normal pancreatic cells (HPDE) clustered away from tumour cell lines. Interestingly, when HPDE cells were grown in a more enriched tumour media (M199/F12 plus additives (described in Additional file 1: Supplementary methods), their metabolic profile appeared closer to - but still distinct from - those of PDCLs. Interestingly, HPDE cultured in M199/F12 also adopted a more mesenchymal morphology (not shown). Three distinct groupings of different PDCLs were apparent, suggesting similarity in metabolite profiles between individual tumours in each of these groups. All PDCLs cultured in low oxygen (TKCC-15-LO, -16-LO, -17-LO, -19-LO, -26-LO) were clustered together, along with TKCC-05, TKCC-10, and TKCC-06. Further, two PDCLs harbouring mutations in ETC complex III (TKCC-03 and TKCC-18-LO) formed a distinct cluster. Importantly, PDCLs did not cluster based on the culture media they were grown in, suggesting that differences in metabolite profiles were not strongly influenced by differences in media composition. Hierarchical clustering of individual metabolite profiles across all cell lines including HPDE (Fig. 3b) revealed five main groupings. As observed in PLS-DA analysis, HPDE cells grown in KSFM media had distinctly different metabolite profiles from tumour cell lines, with TKCC-03 and TKCC-18-LO (both harbouring complex III mutations) forming distinct branches. Other tumour cell lines clustered quite closely together, with two main subgroups apparent.

When abundance of intracellular metabolites was expressed relative to levels in HPDE cells, a number of differences in key metabolites were observed (Fig. 3c). Metabolites that were significantly different across cell lines are coloured red (higher) and blue (lower), while white indicates no significant change (using ANOVA and Tukey’s HSD analysis). There is no clear grouping of PDCLs in line with mtDNA mutational spectra or culture media. However, five main metabolite clusters were apparent. The first, comprising cysteine, tyrosine, and palmitic acid was significantly more abundant in PDCLs compared to normal HPDE cells. A second cluster containing a number of fatty acids was also significantly more abundant in most tumour cell lines. There was a significant cluster of metabolites that were significantly less abundant in PDCLs. Notably, succinate abundance was significantly higher in both TKCC-03 and TKCC-18-LO (Fig. 3d), which harbour non-synonomous somatic mutations in *CytB* (ETC complex III) (Table 1). This effect is entirely consistent with disrupted CytB activity in these cells, which would be expected to impede the conversion of succinate to fumarate. Mullen et al. [54] have previously observed high levels of succinate in cells with ETC complex III mutations using reductive carboxylation.

**Fig. 4:**
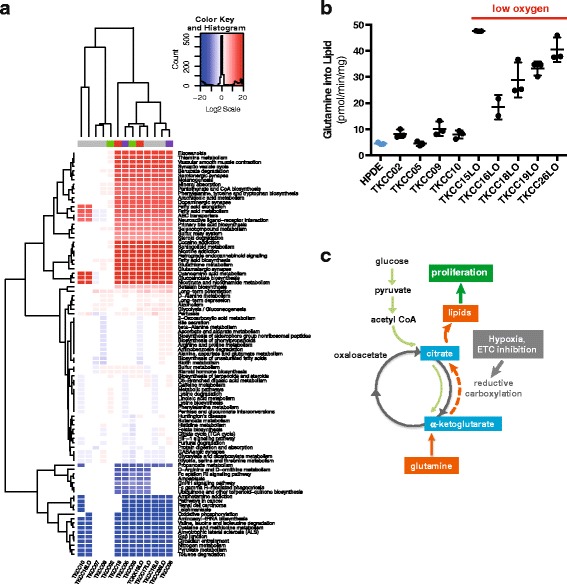
Metabolic pathway flux analysis: **a**. Heirarchical clustering of significantly altered metabolic pathways (ANOVA, *p* < 0.05) in 13 PDCLs relative to normal HPDE cells (in K-SFM media), identified by PAPi analysis. Various culture media are represented by colours along the top of columns (*grey* = M199/F12, *green* = RPMI, *purple* = HPAC modified, *red* = IMDM). **b** Conversion of glutamine to lipid by ^14^C glutamine tracing in PDCLs cultured under normoxic and hypoxic conditions. **c** Metabolomics data are consistent with the activation of reductive carboxylation in pancreatic tumours, driving conversion of glutamine to lipid in the presence of low oxygen and ETC inhibition

The flux of metabolites through their respective pathways is key in understanding the role they play in cancer metabolism and the overall metabolic needs of the cancer cell. To predict which metabolic pathways were dysregulated in pancreatic tumour cells, we performed Pathway Activity Profiling (PAPi) analysis of metabolomics data [42]. Pathway activity scores calculated using this method have been shown to be an accurate predictor for metabolic flux [55], even though there may be redundancy between metabolites, with some being key in several pathways. Activity scores were compared between different cell lines and different growth media conditions and formed the input for hierarchical clustering (Fig. 4). ANOVA was used to determine pathways with significantly different activity (*p* < 0.05) in PDCLs.

Pathway analysis parsed PDCLs into 5 distinct clusters (Fig. 4a). Interestingly, TKCC-02, TKCC-07, and TKCC-09, which all harbour somatic non-synonymous complex I mutations formed a very distinct cluster based on metabolic pathway activity. Three main groupings of metabolic pathways were observed. The first contained pathways that were significantly overrepresented in most of the tumour cell lines. These include several key pathways with potentially important roles in cancer (i.e., biosynthesis, signalling, immunity, etc.) including phenylalanine, tyrosine, and tryptophan biosynthesis; fatty acid metabolism; fatty acid elongation; sphingolipid metabolism (important in biological membranes); eicosanoids (fatty acid-derived signalling molecules important in inflammation and immunity, e.g., prostaglandins) along with its precursor arachadonic acid metabolism; and steroid degradation. Consistent with the Warburg effect, the second branch indicated that glycolysis was upregulated in most of the tumour cell lines. The final branch includes downregulated pathways in the tumour cell lines relative to normal HPDE. Notably, oxidative phosphorylation was significantly attenuated in PDCLs, consistent with mitochondrial dysfunction caused by the somatic mtDNA mutations detected in PDCLs. Other downregulated pathways included D-Arginine and D-ornithine metabolism; ubiquinone and other terpenoid-quinone biosynthesis; and pyruvate metabolism. No consistent effects of either media type or oxygen levels during incubation were observed on clustering.

### Radiolabelled glutamine tracing

Metabolomics analysis strongly indicated upregulated fatty acid synthesis in pancreatic PDCLs (Fig. 4a) and using radiolabelled glutamine tracing we observed significant conversion of glutamine to lipid in these cells (Fig. 4b). Significantly, relatively higher conversion of glutamine to lipid was observed in PDCLs cultured under hypoxic conditions (i.e., TKCC-15-LO, -16-LO, -18-LO, -19-LO, -26-LO). These effects are consistent with previous observations of reductive carboxylation in cells with mitochondrial defects [54], and glutamine dependence in pancreatic tumours with KRAS mutations (Fig. 4c) [9].

## Discussion

A feature of pancreatic cancer, and one of its earliest molecular changes, is a constitutively activating oncogenic KRAS mutation [2, 3]. KRAS signalling in cancer drives cell proliferation and promotes survival. In pancreatic cancer cells, KRAS, along with several other oncogenes (e.g., AKT) and tumour suppressors (e.g., TP53), have been shown to regulate metabolic pathways Jones and Schulze [6]. These metabolic changes include elevated partitioning of glucose carbons to biosynthetic pathways, increased use of glutamine to support growth, and increased NADPH/NADP^+^ ratio to prevent oxidative damage from reactive oxygen species (ROS), maintain cellular redox state, and for use in biosynthesis [9]. Mutant *Kras* copy number has also been shown to result in metabolic reprogramming in vivo in a mouse model of lung cancer, with increased channelling of glucose-derived metabolites into the TCA cycle and glutathione biosynthesis [56]. Understanding the mechanistic basis of these metabolic alterations and their role in tumourigenesis is the focus of intense interest. [6, 11–13] Targeting metabolism as an effector of oncogenic signal transduction pathways required for cell growth may be an effective way of treating cancers that are driven by genetic alterations that are not tractable as direct drug targets [11, 19]. Of direct interest to pancreatic cancers, which have very high penetrance of KRAS mutations, targeting metabolic enzymes has been shown to be effective in treatment of KRAS mutant tumours in pre-clinical models of lung cancer [12].

Mitochondria are the primary site for energy generation within cells and are regulated by interplay between the nuclear and mitochondrial genomes. The mitochondrial genome (mtDNA) encodes 37 genes, including 13 subunits of the mitochondrial electron transport chain (ETC). Mitochondrial dysfunction and/or mutations in mitochondrial genes may play a role in shifting cellular metabolism to a state more favourable for tumour proliferation [20, 21]. Accumulation of somatic mtDNA mutations has been observed in various tumour types [26, 27] and a limited number of studies have shown a direct role for specific mtDNA mutations in tumourigenesis using mitochondrial cybrid models [27–29, 57]. There is evidence that mtDNA mutations affect respiratory complex assembly [58, 59] but in the cancer context, there is limited understanding of genotype-phenotype relationships, or how these mutations precisely impact on mitochondrial function and overall cellular metabolism. Somatic mtDNA mutations have been identified in almost all human cancers, including pancreatic cancer [22, 26, 27]. While earlier studies suggested somatic mutations occur by chance and are likely neutral, more recent evidence suggests these mutations are likely tumourigenic [60]. A 2009 study described mtDNA mutations that alter ROS generation and apoptosis [61] and a 2010 review of 33 studies for 1227 tumour samples reported 50% to carry potentially tumourigenic somatic mtDNA mutations [62].

We identified numerous novel mutations in mtDNA-encoded subunits of the electron transport chain (ETC) in pancreatic cancer (Table 1), with several novel mutations also identified in nuclear encoded mitochondrial genes (Table 3). These mutations were somatic—that is, they were not inherited or found in matched normal tissues but we cannot exclude the possibility that some mtDNA mutations arose secondary to PDCL culture conditions. Every patient cell line tested had at least one mutation, with most harbouring multiple mutations. The majority of the mutations identified were predicted to have a functional effect, either by causing a change in an amino acid coding (the majority located in ETC complexes I, III, or IV), or due to presence in a control region of DNA (Table 2, Fig. 1b). These data are strongly suggestive of positive selection bias for somatic mutations causing functional effects via amino acid changes or transcriptional control (Fig. 1c). Strikingly, we did not observe any somatic mutations in nuclear-encoded ETC subunits across the tumour cohort.

Given the critical role of mitochondria in metabolism, these somatic mutations in tumour cells may be important drivers of deregulated tumour metabolism. This characteristic shift towards increased biosynthesis and aerobic glycolysis, and often a decrease in oxidative metabolism, has been described in other cancers, such as breast cancer [63], but its cause(s) are currently not well described. While other studies have identified somatic mtDNA mutations in cancers [22, 27, 64, 65], in many cases, the functional effects of these variants has not been well characterised. This study is the first to use patient derived cell lines to connect genotype to phenotype in pancreatic cancer by measuring metabolic function in the context of somatic mtDNA mutations.

Somatic variants identified in mitochondrial ETC and metabolic genes in pancreatic tumours were associated with functional effects on metabolic phenotype. Seahorse analysis showed overall decreased oxidative metabolism, and increased glycolysis in all PDCLs (Fig. 2). In particular, PDCLs harbouring non-synonomous mutations in mtDNA ETC complex I genes showed the highest decreases in oxygen consumption (Fig. 2). Mitochondrial mass was previously shown to be increased in metastatic breast cancer [66, 67]. However, measurement of mitochondrial abundance in pancreatic cancer cell lines using flow cytometry showed similar mitochondrial levels across various cell lines (not shown), suggesting that any observed phenotype effects were due to mutations or expression differences in mitochondrial and metabolic genes between cell lines, rather than bulk differences in mitochondrial number.

The apparent heterogeneity of mutations in mtDNA is consistent with that observed in the nuclear genome of pancreatic cancer [31]. However, our data suggest that mitochondrial mutations or genotypes may be categorised into only relatively few metabolic phenotypes. In other words, mutations in different base positions but with similar functional consequences (e.g., complex I dysfunction) exist. Mutations within the same mtDNA gene have previously been shown to result in similar subunit assembly profiles [68]. In addition, a targeted list of over a thousand nuclear-encoded genes encoding mitochondrial proteins and key metabolic proteins was studied in the TKCC patients. This analysis revealed 18 novel somatic mutations across 12 PDCLs. Nearly all of these mutations resulted in amino acid changes (Table 3) in well-known metabolic genes such as LDHB (which is important in glycolysis for the conversion of lactate and pyruvate), TRIT1, which has been proposed as a tumour suppressor [69] and CPT1C, important in fatty acid metabolism. Hence, it is highly likely that mutations in nuclear mitochondrial and metabolic genes are acting in concert with mtDNA mutations to contribute to the metabolic phenotype in pancreatic tumours.

Metabolomics analysis showed some clear common effects in tumours when compared to normal cells (Fig. 3). A recent study used metabolite profiling in concert with gene expression to identify three metabolic subtypes of pancreatic cancer (reduced proliferation, glycolytic, and lipogenic) [70], but a limitation of this study was that it used only cell lines and somatic mutations were not investigated. Overall, there were larger differences between normal cells and tumours than between individual tumours, but variance was also evident between tumours. Three distinct groupings of PDCLs were apparent, suggesting similarity in metabolite profiles between individual tumours in each of these groups. For example, all PDCLs cultured in low oxygen clustered together. Further, two PDCLs harbouring mutations in ETC complex III (TKCC-03 and TKCC-18-LO) formed a distinct cluster. Notably, significantly increased levels of succinate were observed in these lines, as has previously been observed for other tumours harbouring ETC complex III/IV mutations [54]. Importantly, PDCLs did not cluster based on different culture media used, suggesting that differences in metabolite profiles were not strongly influenced by differences in media composition. While changes to individual amino acid abundance in PDCLs are generally consistent with previous observations in murine PDAC [71], it should be noted that tumours from engineered mice with defined, homogeneous genetic background would not necessarily capture the characteristic heterogeneity of human PDCLs used in our study.

Changes in several noteworthy metabolites between normal and tumour cells were identified in metabolomics analysis, implicating activation of reductive carboxylation in pancreatic tumours with mitochondrial mutations. Mullen et al. [72] showed that proliferating malignant prostate cancer cells with complex I or complex III mutations, or even normal mitochondria targeted by other pharmacological mechanisms of inhibiting ETC, were dependant on glutamine as their major source of citrate formation and lipogenic precursor production via reductive rather than oxidative metabolism. These cells are able to use isocitrate dehydrogenase (IDH) to convert α-ketoglutarate to isocitrate, which can then be converted to citrate, and then Acetyl-CoA used in fatty acid and lipid synthesis. Both fatty acid and lipid synthesis are key biosynthetic pathways in cancer proliferation [13, 73] and our pathway analysis (Fig. 4) indicated increased fatty acid synthesis pathways in tumour cells. In the context of mtDNA mutations described above, these metabolomics data are consistent with the activation of reductive carboxylation in the pancreatic tumours studied. Indeed, our observation of conversion of glutamine to lipid in pancreatic PDCLs, and its upregulation in hypoxic conditions (Fig. 4) further supports this assertion. However, we cannot exclude the possibility that glutamine may be providing carbon for lipogenic citrate through its canonical oxidation via the TCA cycle.

**Fig. 5:**
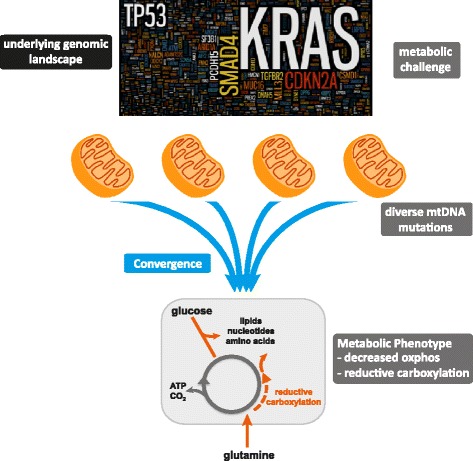
Proposed convergence model of mtDNA mutations driving metabolic adaptation in pancreatic cancer. We propose a model in which the underlying nuclear genomic landscape of pancreatic cancer cells induces a metabolic challenge. As an adaptation to increased biosynthetic requirements, tumours attenuate oxidative phosphorylation through positive selection for diverse somatic mitochondrial mutations, which converge on common metabolic phenotypes

Hence, we propose a model whereby pancreatic tumours attenuate mitochondrial oxidative phosphorylation through positive selection for various somatic mitochondrial mutations, initiating reductive glutamine metabolism to promote the production of biosynthetic intermediates to support cell proliferation (Fig. 5). These observations are consistent with recent gene expression analyses in various cancer types, which indicate that cancer cells converge towards common metabolic landscapes (including downregulation of mitochondrial pathways) via different genetic mechanisms in cancer evolution [74, 75]. That is, there may be positive selection for attenuation of ETC function in pancreatic tumours as an adaptation to increased biosynthetic requirements, consistent with the recently proposed evolutionary model of pancreatic cancer [8]. In this context, it is interesting to note the increased risk of pancreatic cancer associated with diabetes and obesity and that the anti-diabetes drug metformin increases the dependency of prostate cancer cells with normal ETC function on reductive glutamine metabolism [76].

## Conclusions

Previous studies have implicated mutations or altered expression in individual enzymes, or alterations in specific signalling pathways (e.g., PI3K, AKT or mTOR) in driving the metabolic adaptations observed in a variety of cancers [16, 17, 77–79]. In contrast, we propose a highly novel mechanism for the metabolic shift observed in pancreatic tumours. In short, the heterogeneous genomic landscape of pancreatic tumours may converge on a common metabolic phenotype, with individual tumours adapting to increased anabolic demands via different genetic mechanisms (Fig. 5). Hence, targeting common phenotypic traits, rather than specific genetic lesions, may be a productive therapeutic strategy in pancreatic cancer.

## Additional files

Additional file 1:
**Figure S1**: Sanger sequencing traces from matched normal, primary tumour and PDCL for TKCC-10, showing the presence of a somatic heteroplasmic insertion in primary tumour, which progresses to homoplasmy in the PDCL. **Table S1**: Nuclear mitochondrial and metabolic genes analysed for somatic mutations. **Table S2**: Detailed description of function in nuclear mitochondrial and metabolic genes with non-synonymous mutations in PDCLs. **Table S3**: Intracellular metabolites (*n*=72) targeted in metabolomics analaysis. (PDF 990 kb)
Additional file 2:
**Table S4**: Metabolomics data. (XLSX 56 kb)

## Abbreviations

APGI: Australian Pancreatic Cancer Genome Initiative
ECAR: Extracellular acidification rate
ETC: Electron transport chain
HPDE: Human pancreatic ductal epithelia
mtDNA: Mitochondrial genome
OCR: Oxygen consumption rate
OxPhos: Oxidative phosphorylation
PAPi: Pathway activity profiling
PDAC: Pancreatic ductal adenocarcinoma
PDCL: Patient-derived cell line
PDX: Patient-derived xenograft
TCA: Tricarboxylic acid
