# Supplementary material for: Mitochondrial mutations and metabolic adaptation in pancreatic cancer
Source: Cancer Metab. 2017 Jan 30;5:2. doi: 10.1186/s40170-017-0164-1 (PMC5282905; doi:10.1186/s40170-017-0164-1)
Supplement: Additional file 1: — Figure S1: Sanger sequencing traces from matched normal, primary tumour and PDCL for TKCC-10, showing the presence of a somatic heteroplasmic insertion in primary tumour, which progresses to homoplasmy in the PDCL. Table S1: Nuclear mitochondrial and metabolic genes analysed for somatic mutations. Table S2: Detailed description of function in nuclear mitochondrial and metabolic genes with non-synonymous mutations in PDCLs. Table S3: Intracellular metabolites (n=72) targeted in metabolomics analaysis. (PDF 990 kb) [file 40170_2017_164_MOESM1_ESM.pdf]

Supplementary data

Supplementary Figure 1:
Sanger sequencing traces from matched normal, primary tumour and PDCL for TKCC-10, showing the presence of a somatic heteroplasmic insertion in primary tumour, which progresses to homoplasmy in the PDCL.

Supplementary Table 1: Nuclear mitochondrial and metabolic genes analysed for somatic mutations.

Supplementary Table 2: Detailed description of function in nuclear mitochondrial and metabolic genes with non-synonymous mutations in PDCLs.

Supplementary Table 3: Intracellular metabolites (n=72) targeted in metabolomics analaysis.

Supplementary Methods

1. Culture Medium for TKCC-02, 03, 04.

RPMI 1640 (Gibco®)	500 ml
Fetal Bovine Serum (FBS) (HyClone)	10%
hEGF (Invitrogen) (1 mg/ml)	20 ng/ml
Penicillin Streptomycin (Gibco®)	5 ml
(+5,000 U/ml Pen, +5,000 mg/ml Strep)	
Gentamicin (Pfizer) (40 mg/ml)	0.02 mg/ml

2. Culture Medium for TKCC-07, 08, 09, 10, 15-LO, 16-LO, 17-LO, 26-LO.

M199/F12 (HAM) 1:1 (Gibco®)	500ml
HEPES (Gibco®) 1 M	15 mM
L-glutamine (Gibco®) 200mM	2 mM
hEGF (Invitrogen) 1 mg/ml	20 ng/ml
Hydrocortisone 1 mg/ml	40 ng/ml
Transferrin (Sigma) 2.5 mg/ml	5 ug/ml
Insulin (ActRapid) 100 IU/ml	0.2IU/ml
Glucose (Sigma) 10%	0.06%
FCS (HyClone)	7.5%
Tri-iodothyronine (Sigma) 0.1 mg/ml	0.5 pg/ml
MEM vitamins (Gibco®) (100x)	5 ml
O-phosphoryl ethanolamine (Sigma) (20 mg/ml)	2 ug/ml
Penicillin Streptomycin (Gibco®)	5 ml
Gentamicin (Pfizer) (40 mg/ml)	0.02 mg/ml

3. Culture Medium for TKCC-05, 06 (HPAC Modified Medium).

DMEM/F12 (HAM) (Gibco®)	500ml

HEPES (Gibco®)	15 mM
Transferrin (Sigma) 2.5 mg/ml	5 ug/ml
FCS (HyClone)	7%
Insulin (ActRapid) (100 IU/ml)	0.1 IU/ml
hEGF (Invitrogen) 1 mg/ml	10 ng/ml
Hydrocortisone 1 mg/ml	40 ng/ml
Glucose Sigma (10%)	0.12%
Penicillin Streptomycin (Gibco®)	5 ml
Gentamicin (Pfizer) (40 mg/ml)	0.02 mg/ml

4. Culture Medium for TKCC-18-LO, 19-LO (IMDM Modified Medium).

IMDM (Gibco®)	
FCS (HyClone)	20%
hEGF (Invitrogen) 1 mg/ml	20 ng/ml
Transferrin (Sigma) 2.5 mg/ml	2.5 ug/ml
Insulin (ActRapid) 100 IU/ml	0.2IU/ml
MEM vitamins (Gibco®) (100x)	2.5 ml
Penicillin Streptomycin (Gibco®)	5 ml
Gentamicin (Pfizer) (40 mg/ml)	0.02 mg/ml

5. Primers used for mtDNA sequencing

Amplicon 1

Amp1-F	5’-GGCTTTCTCAACTTTTAAAGGATA-3’
12899-R	5'-CATGCTAAGGCGAGGATGAA-3'
12834-F	5'-AGCAGCCATTCAAGCAATCC-3'
13306-F	5'-CTAGCATTCCTGCACATCTG-3'
13588-F	5'-CTGACAAGCGCCTATAGCAC-3'
14447-R	5'-AGGAGTATCCTGAGGCATGG-3'
14371-F	5'-TCCTACCTCCATCGCTAACC-3'
15087-F	5'-ACATCGGCATTATCCTCCTG-3'
15759-F	5'-TCGGAGGACAACCAGTAAGC-3'
16492-F	5'-ATCCGACATCTGGTTCCTAC-3'
42-R	5'-AGAGCTCCCGTGAGTGGTTA-3'
891-R	5'-GGCACGAAATTGACCAACC-3'
751-F	5'-ACAAGCATCAAGCACGCAGC-3'
1721-R	5'-GGCTAAGGTTGTCTGGTAGT-3'
1551-F	5'-GGAGACAAGTCGTAACATGG-3'
2324-F	5'-TTCTCCTCCGCATAAGCCTG-3'

Amplicon 2

2909-F	5’-GACCAACGGAACAAGTTACC-3’
3106-R	5’-GTAGATAGAAACCGACCTGG-3’
3322-F	5’CTCCTACTCCTCATTGTACC-3’
3340-R	5’-GTACAATGAGGAGTAGGAGG-3’
3559-R	5'-GTAGAAGAGCGATGGTGAGA-3'
4162-R	5'-TGAGTTGGTCGTAGCGGAAT-3'
4040-F	5'-CAACATATGACGCACTCTCC-3'
4344-F	5’-TCGAACCCATCCCTGAGAAT-3’
4838-F	5'-TCTGACATCCGGCCTGCTTC-3'
5603-F	5'-CCACTCTGCATCAACTGAAC-3'
5911-F	5’-CCGACCGTTGACTATTCTCT-3’
5995-R	5’-GTGCCTAGGACTCCAGCTCA-3’
6325-F	5'-CCTCCGTAGACCTAACCATC-3'
6413-R	5’ATTGATAATTGTTGTGATGA-3’
6946-F	5'-TCACCGTAGGTGGCCTGACT-3'
7400-F	5’-CCCACCCTACCACACATTCG-3’
7743-F	5'-CTAACATCTCAGACGCTCAG-3'
8254-F	5’-CGTATTTACCCTATAGCACC-3’
8782-R	5'-CGAGGAGGTTAGTTGTGGCA-3'
8642-F	5'-ACAACCGACTAATCACCACC-3'
9001-F	5’-CGCCTAACCGCTAACATTAC-3’
9378-F	5'-TGGCGCGATGTAACACGAGA-3'
10380-R	5'-GTAGTCACTCATAGGCCAGA-3'
10131-F	5'-CCACAACTCAACGGCTACAT-3'
10534-F	5'-ATCGCTCACACCTCATATCC-3'
10961-F	5'-CTAACTACCTGACTCCTACC-3'
11680-F	5'-AAGCTTCACCGGCGCAGTCA-3'

Supplementary Figure 1 - Hardie et al

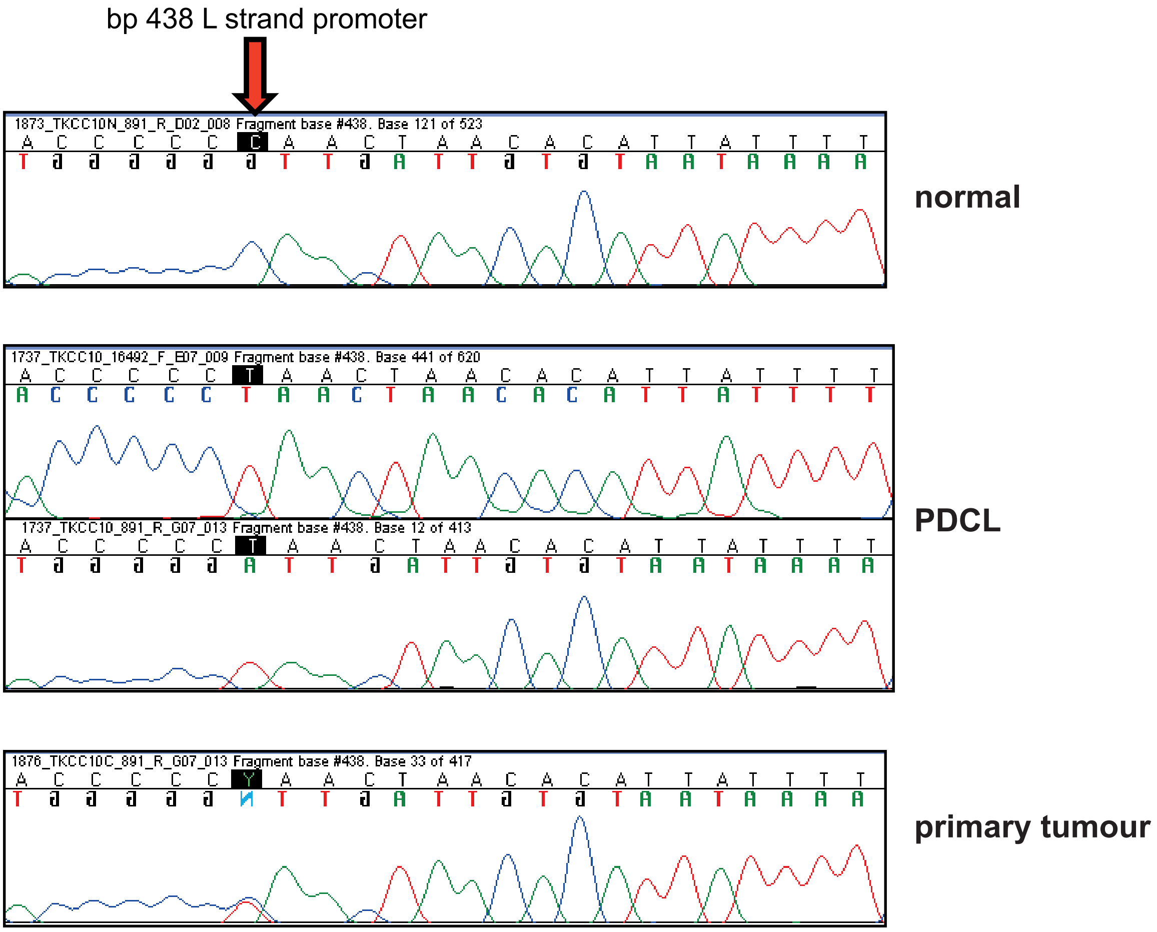

Supplementary Table 2: Detailed description of function in nuclear mitochondrial and metabolic genes with non-synonymous mutations in PDCLs.

Gene mutated	Name and function of protein encoded	Cell line
**TRIT1**	tRNA isopentenyl transferase, lung cancer tumour suppressor (Spinola et al. 2005).	TKCC-02
**TP53**	Tumour protein 53, regulates cell cycle and acts as tumour suppressor (Cho et al. 1994).	TKCC-04, TKCC-17-LO (different site)
**ACACA**	Acetyl Co-A carboxylase, involved in rate limiting step of fatty acid synthesis.	TKCC-04
**CPT1C**	Carnitine palmitoyltransferase I, an acetyltransferase involved in transport of long chain fatty acids across outer mitochondrial membrane by binding them to carnitine. Important in fatty acid metabolism.	TKCC-04
**PIK3CA**	Phosphatidylinsitol 3-kinase, signalling important in many cancers, eg. Mutation associated with improved colorectal cancer survival with adjuvant aspirin therapy (Liao et al. 2012).	TKCC-04
**PDHA2**	Pyruvate dehydrogenase (lipoamide) alpha 2, a testis-specific mitochondrial matrix enzyme that catalyses the oxidative decarboxylation of pyruvate, producing acetyl-CoA and CO_2. Key enzyme in controlling the balance between lipid and glucose oxidation depending on substrate availability.	TKCC-04
**AKT3**	RAC-gamma serine/threonine-protein kinase, enzyme, AKT kinases are regulators of cell signalling in response to insulin and growth factors. This kinase has been shown to be stimulated by platelet-derived growth factor (PDGF), insulin, and insulin-like growth factor 1 (IGF1).	TKCC-09
**YME1L1**	ATP-dependent metalloprotease YME1L1, gene proposed to plays a role in mitochondrial protein metabolism and could be involved in mitochondrial pathologies (Coppola et al. 2000).	TKCC-09
**ADCK4**	aarF domain containing kinase 4, This gene encodes a protein with two copies of a domain found in protein kinases. The encoded protein has a complete protein kinase catalytic domain, and a truncated domain that contains only the active and binding sites of the protein kinase domain, however, it is not known whether the protein has any kinase activity.	TKCC-09, TKCC-16-LO (different site)
**MTCP1**	Protein p13 MTCP-1, oncoprotein (Yang et al. 1998).	TKCC-15-LO
**HMGCL**	3-Hydroxymethyl-3-Methylglutaryl-CoA Lyase, mitochondrial enzyme that catalyzes the final step of leucine degradation and plays a key role in ketone body formation. Mutations in this gene are associated with HMG-CoA lyase deficiency.	TKCC-16-LO
**TARS2**	Threonyl-TRNA Synthetase 2, Mitochondrial (Putative), mitochondrial aminoacyl-tRNA synthetase.	TKCC-18-LO
**LDHB**	Lactate dehydrogenase B, enzyme which catalyzes the reversible conversion of lactate and pyruvate, and NAD and NADH, in the glycolytic pathway. Mutations in this gene are associated with lactate dehydrogenase B deficiency. Implicated in cancers (McCleland et al. 2012; Brown et al. 2013)	TKCC-26-LO
